# Donor HLA−DR Drives the Development of De Novo Autoimmunity Following Lung and Heart Transplantation

**DOI:** 10.1097/TXD.0000000000001062

**Published:** 2020-09-24

**Authors:** Ewa Jankowska−Gan, Vrushali V. Agashe, Diego A. Lema, Ying Zhou, Laura Gonzalez Bosc, Jeremy A. Sullivan, Daniel S. Greenspan, William J. Burlingham

**Affiliations:** 1 Department of Surgery, Division of Transplantation, School of Medicine and Public Health, University of Wisconsin−Madison, Madison, WI.; 2 Department of Cell Biology and Physiology, University of New Mexico Health Sciences Center, Albuquerque, NM.; 3 Department of Cell and Regenerative Biology, School of Medicine and Public Health, University of Wisconsin−Madison, Madison, WI.

## Abstract

Individuals harbor preexisting HLA−DR/DQ−restricted responses to collagen type V (ColV) mediated by Th17 cells under Treg control, both specific to peptides that bind to inherited HLA class II antigens. Yet after transplant, the donor−DR type somehow influences graft outcome. We hypothesized that, long after a lung or heart allograft, the particular HLA−DR type of the mismatched transplant donor transforms the specificity of the “anti−self” response. This could explain why, over long term, certain donor DRs could be more immunogenic than others.

**Methods.:**

We analyzed 7 HLA−DR15^neg^ patients who had received a lung allograft from a DR15^+^ donor. To determine the mechanism of acquired specificity in self−reactivity, we analyzed the kinetics of DR1 (host) and DR15 (donor) peptide restriction in a heart transplant model using DR−transgenic mice.

**Results.:**

Beyond 1.5 years post-lung transplant, all patients tested had acquired DR15−restricted immune responses to ColV peptides. These responses were either unrestrained Th17 type (n = 4) or Th17 controlled by Treg arising early (<5 y) or late (>7 y) after transplant (n = 4). Treg suppression via conventional (transforming growth factor−β [TGF−β]) and extracellular vesicle−associated (IL−35) cytokines correlated with superior outcomes. Naïve DR1 and DR15 transgenic mice had preexisting DR−restricted responses, exclusively to ColV fragments containing DR1− or DR15−binding peptides. When HLA−DR1 transgenic recipients of a HLA−DR15 heart developed ColV reactivity post-transplant, mice that acutely rejected (20–25 d) responded only to the DR1−restricted ColV peptide epitope. In animals whose grafts survived long term, we could detect acquisition of DR from the transplant donor onto the surface of recipient dendritic cells, and immune responses against a donor DR15–restricted ColV peptide.

**Conclusions.:**

These results might explain how certain donor HLA−DR types redirect host immune responses to novel peptides of critical self−antigens. Unless regulated, such responses may predispose the allograft to chronic rejection.

## INTRODUCTION

Lung transplantation is the only treatment option for patients with end−stage lung disease.^1^ Although short−term survival has improved, the greatest challenge to long−term graft survival is the development of chronic lung allograft dysfunction,^2,3^ manifesting as bronchiolitis obliterans syndrome (BOS), causing the 5−year survival rate to remain low at 50%. We previously reported that the development of a Collagen type V (ColV)−specific IL−17−dependent cellular immune response after lung transplantation was strongly correlated with BOS.^4^ Similarly, the development of antibody and T−cell responses to another key “self” antigen, ĸ−α1−tubulin was also shown to contribute to lung allograft chronic rejection.^5^

Th17−mediated self−reactivity to ColV is occasionally observed *before* lung transplantation in connection with preexisting pulmonary disease and constitutes a significant risk factor for primary graft dysfunction.^6^ Furthermore, HLA analysis of patients with immune responses to ColV before transplant revealed that such individuals were more likely to express HLA−DR15,^7^ suggesting that certain DR types predispose to greater autoimmunity to ColV.

We previously reported that Th17−dependent responses to ColV are normally kept in check by CD39^+^ Treg.^8^ ColV is one of a trio of self−antigens, including ĸ−α1−tubulin and vimentin, to which neutralization of TGF−β, IL−35, CD39 enzymatic activity, or the depletion of Treg, can uncover Th17 responses in healthy individuals. These “natural” Th17 responses arise during fetal life, are self−HLA−DR/DQ−restricted, and can be induced by large (~160 aa), but not by small (15aa), ColV α1 chain fragments that contain the DR−specific peptide.^8^ This is presumably due to a requirement for a “second signal” via LAIR1 binding to repetitive glycine−proline−hydroxyproline sequences within the larger fragment.^9,10^

Of all the HLA−DRs tested so far, the greatest ColV peptide binding activity was seen for HLA−DR15. This was consistent with high levels of endogenous (Treg−controlled) ColV reactivity in DR15^+^ individuals.^7,8^ Before lung transplantation, those end−stage lung disease patients who were HLA−DR15^+^ tended to be among those who had lost Treg control, and were significantly more ColV−responsive and subject to primary graft dysfunction, than were DR15^neg^ patients.^6,7^ On the other hand, those patients who were DR15^neg^ but developed ColV reactivity after transplant, were more likely to have a lung *donor* that expressed HLA−DR15.^7^ DR15^neg^ recipients of DR15^+^ lung transplants went on to develop BOS at a higher frequency.^11^

Previously, we showed that a HLA−DR15^neg^ recipient (DR1, 11) who had received a lung allograft from a DR1,15 donor, developed a strong immune response to the DR15−restricted ColV α1 chain peptide p1049 at 7 years post-transplant.^7^ To see if this was a common feature of DR15^+^ →DR15^neg^ lung transplants, we extended our study to examine the influence of HLA−DR15 expressed by the lung transplant donor on the immune response to ColV and ColV−specific peptides in 6 additional HLA−DR15^neg^ recipients. We also revisit that original patient^7^ at 14 years post-transplant. Furthermore, to determine the time course of the change in HLA−DR restriction of the host response to ColV after an allograft, we investigate here the role of donor DR in a murine heterotopic heart transplant model using HLA−DR−transgenic mice. Just as major histocompatibility complex (MHC)−class II acquisition from the mother required uptake by neonatal dendritic cells (DC) of maternal extracellular vesicles (EV),^12^ the results presented here support the concept of acquisition of HLA−DR molecules from EV of the transplant donor delivered to host dendritic cells.

## MATERIALS AND METHODS

### Human Subjects

Immunological monitoring was performed on samples obtained from lung transplant recipients according to informed consent procedures, subject to human subjects Institutional Review Board approval at the University of Wisconsin−Madison. All were HLA−DR15^neg^ patients, who had received a lung allograft from a DR15 donor, including one whose self/donor−shared (DR1−restricted) and donor DR15–restricted immune responses to ColV at 7 years post-transplant has been described previously.^7^

### Peripheral Blood Mononuclear Cells Isolation

Peripheral blood mononuclear cells (PBMC) were obtained by venipuncture and collected into acid citrate dextrose tubes (Becton−Dickinson, Franklin Lakes, NJ) and further purified using Lymphocyte Separation Medium (Corning, Mediatech Inc., Manassas, VA). PBMC were washed 2–3 times in Dulbecco’s PBS (DPBS; Corning, Mediatech Inc.) to reduce platelet contamination.

### Mice

C57BL/6 (B6) mice were purchased from Envigo (Indianapolis, IN). CB17 severe combined immunodeficient (SCID) mice were obtained from the UW−Madison Mouse Breeding Core. Major histocompatibility complex class II−deficient mice expressing HLA−DRB1*1501.AE° (DR15) were provided by Dr Chella David (Mayo Clinic, Rochester, MN)^13^ and the HLA−A2.01/HLA−DRB1*0101.AE° (A2/DR1) transgenic mice were provided by Dr François Lemonnier (Institut Pasteur, Paris, France).^14^ The DR15 mice were extensively back crossed onto an IAb^−/−^ B6 background in−house. Male and female mice were used in experiments. All animals were housed in a specific pathogen−free facility, and experiments were conducted in accordance with the NIH guidelines and after approval of the Institutional Animal Care and Use committee. Animals were euthanized at a specific time point or at the time of graft rejection.

Spleen and lymph nodes were collected, and single−cell suspensions were prepared for experimental use. To obtain graft−infiltrating lymphocytes, the native and graft hearts were collected, organs were cut into smaller pieces and incubated with Collagenase IV (600 U/mL) and DNase I (60 U/mL) in HBSS for 30 minutes at 37 degrees. The tissue was then mashed through a fine mesh screen (Corning Cell Strainer 100 µm Nylon) and washed with DPBS to get a single−cell suspension for experimental use.

### Trans-vivo Delayed Type Hypersensitivity Assay

Trans-vivo delayed type hypersensitivity (Tv−DTH) assay was performed by cotransfer of 7–10 × 10^6^ human PBMCs or 10 × 10^6^ mouse splenocytes with appropriate antigens into the footpads of SCID mice as previously described.^7, 15^ Tetanus toxoids and diphtheria toxoid (Sanofi Pasteur Inc., Toronto, Ontario, Canada) recall antigen alone plus cells (PBMCs or spleen) were used as a positive control, with cells plus PBS as a negative control. Antigen−driven swelling was measured after 24 hours using a dial thickness gauge. Pre-injection and post-injection measurements were compared, and DTH reactivity is shown as the change in thickness, using units of 10^−4^ inches over background swelling due to cells plus PBS alone.

For human studies, 5 μg of ColV or ColI protein or 1 μg of ColV peptides were injected. To determine the cytokine involvement in these responses, 25 µg of neutralizing antibodies to TGF−β or Rabbit IgG control or to subunits of IL−35 (IL−12αp35, Ebi3, 1 µg of each) were included in the assay.

For murine studies, 20 μg of ColV and ColI protein were injected. In the case of peptides, ColV fragments and vimentin, 5 μg were administered. Donor− and self−antigens were prepared from splenocytes by sonication as described previously.^15^ The disrupted cells were centrifuged for 20 minutes at 14 000 g to remove membrane debris. The protein content of the supernatant was determined using standard methods. Ten micrograms of protein were used for each injection.

To determine the effect of neutralizing antibodies, spleen cells were mixed with the antigen of interest and injected into the footpads of SCID mice with 10 µg of either control IgG or anti-mouse TGF−β1.

### Sources of Antigens and Antibodies

ColI and ColV (Human and Bovine) were a gift from Dr David Wilkes (UVA, Charlottesville, VA) or Dr David Brand (University of Tennessee, Memphis, TN). ColV fragments were produced and purified in−house (see Table S1, SDC, http://links.lww.com/TXD/A285), as described previously.^16^ Vimentin was purchased from R&D Systems (Minneapolis, MN). ColV peptides were obtained from Genscript (Piscataway, NJ), or the UW peptide synthesis core.

Neutralizing antibody to TGF−β (AB−100−NA) and Normal Rabbit IgG Control (AB−105−C) were purchased from R&D Systems (Minneapolis, MN). Purified rat anti-mouse TGF−β1 (Cat555052), and rat IgG1ĸ isotype control were purchased from BD Bioscience (San Jose, CA). Anti−hIL12/IL−35 p35 (MAB15701) was purchased from R&D (Minneapolis, MN).

Anti−Ebi3 was a gift from Dr Dario Vignali, University of Pittsburgh.

### Murine Organ Transplantation

All transplants were between the HLA−DR−transgenic mice (on a B6 background) or B6 mice, as described. Heterotopic cardiac transplantation was performed intra-abdominally using the technique described by Corry et al^17^ and modified by simplifying the anastomosis.^18^

Eight to 10−week−old age−matched donor and recipient male mice were used for transplantation.

Briefly, recipient mice were anesthetized with inhaled isoflurane. Proximal and distal ties with 6−0 silk were placed around the aorta and vena cava. A midline abdominal incision was made in the donor, and 10% heparin was injected into the inferior vena cava. Superior and inferior vena cavas were ligated with 6−0 silk and divided superior and inferior to the tie, respectively. Blood was evacuated from the heart, and pulmonary veins were ligated and divided distal to the ligature. The heart was lifted from the chest and placed in chilled Ringer’s lactate solution.

The recipient was clamped around the aorta and vena cava. The donor aorta and pulmonary artery were joined end−to−side to the recipient aorta and vena cava. Clamps were removed, and transplants perfused with oxygenated recipient blood.

Grafts were monitored daily by abdominal palpitation as previously described.^19^

### Graft Survival

HLA−DR15 transgenic cardiac grafts could be divided into 2 groups based on graft survival: (a) grafts that were rejected, by day 20 post-transplant, were classified as acute rejectors, while (b) those that survived long term (days 80–100 post-transplant) were classified as chronic rejectors. Three grafts that survived to intermediate lengths (days 20–80) were not included in the analysis.

### Flow Cytometry

1 × 10^6^ cells were Fc blocked (Biolegend101320) and stained for 1 hour, on ice, in the dark, as previously described.^20^ The following antibodies were used, B220−FITC (BD553087), I Ab PerCP−Cy5.5 (Biolegend116416), Ly6C PE or PECy7 (Biolegend128008, 128071), HLA−DR PECy7 (eBioscience25−9956−41/Biolegend307606), CD11b BV510 (BD562950/Biolegend101263), Ly6G BV605 (BD563005/Biolegend127639), CD11c APC (BD550261), CD3 A700 (eBioscience56−0032−82/Biolegend100216), Live Blue Fluorescent reactive dye (LifeTech L34962), and CD45 BV650 (Biolegend103151).

### Statistics

Data were analyzed using GraphPad Prism software (La Jolla, CA). The *P* values were calculated using Mann−Whitney *U* tests: *P* < 0.05 was considered a significant difference.

## RESULTS

### Development of Donor HLA−DR−restricted Anti−ColV Responses After Lung Transplantation

**FIGURE 1.**
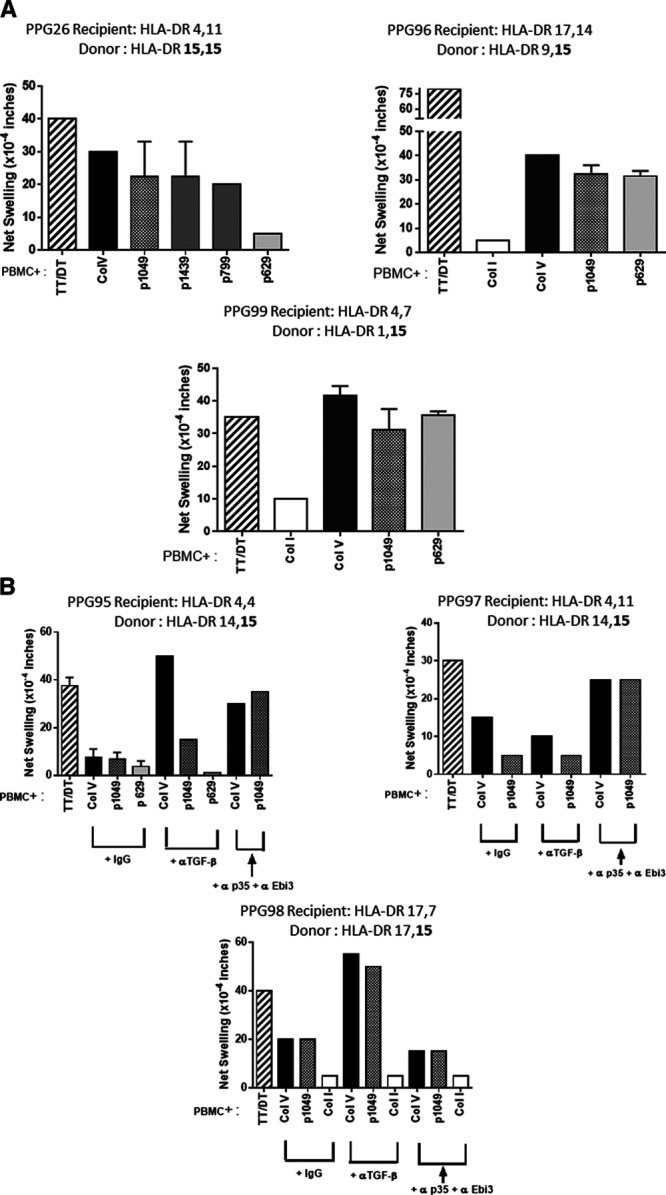
Donor HLA−DR15 results in 2 types of DR15−restricted responses to ColV post-lung transplantation. A, Development of *unrestrained* donor HLA−DR15−restricted autoimmune responses to ColV in DR15^neg^ patients ≥1.5 y after lung transplant. Three patients who had received a graft from a DR15^+^ donor were analyzed by Tv−DTH. PPG26 developed responses to the DR15−restricted peptide p1049 as well as to the DR1/15 cross-reactive peptides p799 and p1439. No response to the DR1−restricted ColV peptide p629 was noted. In DR15^neg^ patients PPG96 and PPG99, we also found strong responses both to ColV and to the DR15−restricted peptide p1049. PPG99 also had a strong response to the donor DR1–restricted ColV peptide p629. PPG96 also responded to p629; whether this is due to peptide presentation by the donor DR is uncertain. B, Development of *regulated* donor DR15–restricted responses to ColV in DR15^neg^ patients ≥1.5 y after lung transplantation. PPG95, PPG97, and PPG98 also received a lung allograft from a DR15^+^ donor. While their PBMC did not demonstrate any ColV or ColV peptide reactivity, post-transplant neutralization of TGF−β (PPG98), IL−35 (PPG97), or both (PPG95) revealed a regulated response to ColV as well as the DR15−restricted peptide p1049 post-transplant. PBMC, peripheral blood mononuclear cells; TGF−β, transforming growth factor−β; Tv−DTH, trans-vivo delayed type hypersensitivity.

In order to investigate the association between HLA−DR15 expression by lung transplant donors and expanded immune responses to ColV in recipients, we analyzed a group of lung transplant recipients 1.6–14 years after transplantation, who were HLA−DR15 *negative* but had received a transplant from an HLA−DR15^+^ donor (Table 1). Based on their PBMC response to ColV in the Tv−DTH assay, the patients could be divided into 2 groups. In the first group, patients PPG26, PPG96, PPG99 responded to whole ColV, as well as to the DR15—associated peptide p1049 (Figure 1A). In the case of PPG26, response was also seen to the HLA−DR1/15 cross-reactive peptides p799 and p1439, much like in the previously described patient L86.^7^ The HLA−DR1 binding peptide p629 also induced a high Tv−DTH swelling response in patients PPG96 and PPG99. Together, these findings support the previous observation in L86 at year 7 post-transplant that HLA−DR15 expressed by the lung transplant donor transforms the immune response to the self−antigen ColV in a HLA−DR15^neg^ recipient, allowing a response to both a DR1−restricted epitope (p629), and also to the p1049 epitope via donor DR15.

**TABLE 1.** Demographics and clinical characteristic of patients

Patient ID	Recipient HLA-DR	Donor HLA-DR	Time post-transplant (y)	Age	Gender	Clinical outcome (at time of testing)
PPG26	4, 11	15, 15	1.6	46	Male	Some element of BOS
PPG96	17, 14	9, 15	2	55	Female	Presumed antibody−mediated acute rejection treated with pulse steroids at 1 y
PPG99	4, 7	1, 15	3.1	68	Male	Signs of increased obstruction including a decreased ratio and significant change in FEV1
PPG95	4, 4	14, 15	1.7	67	Male	Stable with very good pulmonary graft function, FEV1 103%
PPG97	4, 11	14, 15	2.7	69	Male	Stable graft function, FEV1 107%
PPG98	17, 7	17, 15	4.2	63	Male	Stable with very good pulmonary graft function
L86	1, 11	1, 15	14	69	Male	Stable graft function at the time of testing

BOS, bronchiolitis obliterans syndrome.

The second group of patients (PPG95, PPG97, and PPG98) failed to show proinflammatory responses to ColV. However, when patients PBMC were stimulated by ColV or ColV peptides and injected into the footpads of SCID mice with antibodies neutralizing either conventional (TGF−β) or EV−associated (IL−35)^21^ cytokines, responses to both ColV, and the donor DR15–associated peptide p1049 were revealed (Figure 1B).

Interestingly, there was no third group of patients; that is, patients that failed to respond in some way to ColV peptide p1049, thus indicating that acquisition of a donor DR15–restricted immune response was *universal* in our patient sample.

**FIGURE 2.**
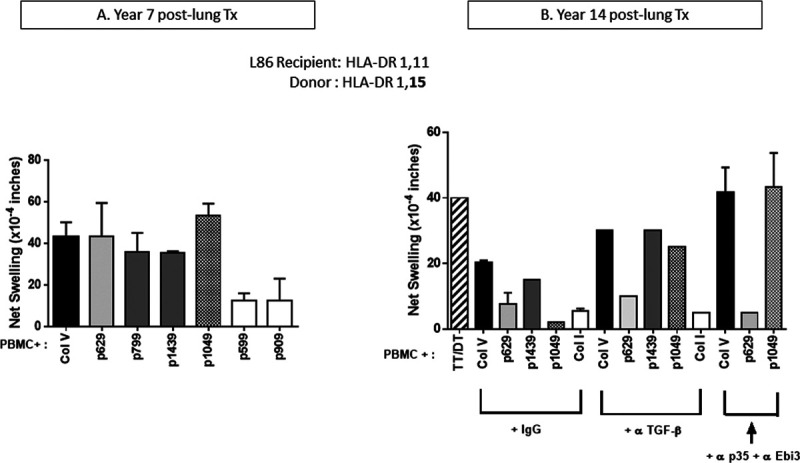
Longitudinal analysis of the development of donor DR15–restricted ColV autoimmunity and its regulation. Patient L86 (HLA−DR1,11) received a lung transplant from a HLA 1,15 donor. Note the strong anti−ColV response at year 7, including a strong donor DR15–restricted response to p1049, as well as a DR1−restricted response to p629 as published previously (7). Tv−DTH responses in the same patient were reanalyzed at 14 y post-transplant. The recipient now had low responses to ColV and p1439 and no response to p1049. But neutralization of TGF−β and IL−35 led to an increase in responses to ColV and p1439 and uncovered a response to the DR15−restricted p1049 but not to the self (DR1)−restricted p629. PBMC, peripheral blood mononuclear cells; TGF−β, transforming growth factor−β.

We previously described another DR15^neg^ patient, who had received a lung allograft from a DR15 donor. He developed both self/donor−shared (DR1−restricted) and donor DR15–restricted immune responses to ColV at 7 years post-transplant^7^ (Figure 2A). We wished to see if these responses persisted, or if over time the acquired response become regulated, as in the case of autologous ColV reactivity. When the same recipient was analyzed again at 14 years post-transplant, the patient responded weakly to ColV and to HLA−DR15 (donor)−restricted p1049 peptide, as well as to the “shared” peptide (ie, 1 bound by both self DR1 and donor DR15) p1439 (Figure 2B). As was the case in normal ontogeny, at this late time point, neutralization with anti−TGF−β or anti−IL−35, uncovered a strong response to ColV. Interestingly, Treg control extended to both p1439 as well as to the donor DR15–restricted peptide p1049 (Figure 2B). Surprisingly, no response to the self/graft shared HLA DR1−restricted peptide p629 was seen at this time point, suggesting that all ColV reactivity at 14 years post−Tx was now entirely restricted to the mismatched donor HLA−DR type. These results support the idea that, just as maternal MHC class II can cause development in newborn mice of PD−L1−dependent tolerance via an exosome secretion/uptake mechanism,^12^ the lung transplant donor DR not only changes the recipient immune response post-transplant, but can also induce the development of a donor DR–restricted immunity, and possibly, immune regulation.

### HLA DR−restricted Natural Responses to ColV Are Detected in DR1 and DR15 Transgenic Mice

Since DR−restricted responses to self−antigens including ColV have been noted in healthy individuals,^8^ we first determined if such DR−restriction was also prevalent in the IAb^−/−^ HLA−DR1 and DR15 transgenic mice. TGF−β1 neutralization in splenocyte preparations from naïve A2/DR1 transgenic mice revealed responses to ColV (Figure S1A, SDC, http://links.lww.com/TXD/A285) and to the ~140 aa fragment 1 of the ColV α1 chain, which contains the DR1−restricted peptide p629, as well as the DQ−restricted peptide 599, but not to fragment 4, which contains the DR15−restricted peptide p1049. Importantly, similar to the human data,^8^ in naïve T−cell responses were noted only to large α1 fragments but not when the cells were stimulated with the DR1−binding ColV 15−mer p629 peptide alone.

Spleen cells from naïve DR15 transgenic mice revealed a relatively high−background response to ColV. The neutralization of TGF−β1 further increased this response to ColV (Figure S1B, SDC, http://links.lww.com/TXD/A285). In contrast to DR1 transgenic mice, spleen cells from DR15 transgenic mice responded to ColV α1 chain fragment 4 (~160mer), containing the DR15−restricted peptide p1049, but not to fragment 1, or fragment 3, which contains the DQ2/IA^b^−restricted peptide p909. These results substantiated the purely HLA DR−restriction of natural ColV responses in HLA−DR transgenic, I−A null mice.

### HLA−DR1 Tg Mouse Recipients of DR15 Tg Heart Allografts Develop Different DR−restricted Immune Responses to ColV During Acute Versus Chronic Rejection

**FIGURE 3.**
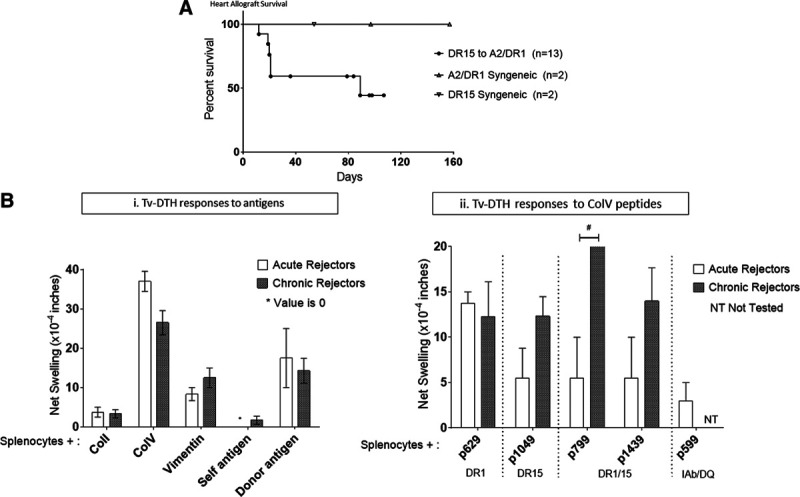
HLA−DR15 (donor)−restricted immune responses develop in transgenic mice with chronically rejecting, but not acutely rejecting, DR−transgenic murine cardiac grafts. A, Naïve HLA A2/DR1 transgenic mice received heterotopic cardiac transplants from HLA−DR15 transgenic donors. Two mice whose grafts were still beating, at a very low score, were sacrificed at day 19 and day 36 post-transplant, and 1 graft survived to day 89. All others survived long term, with evidence of chronic rejection. All 4 syngeneic grafts were accepted and survived long term. B(i), A2/DR1 recipients of the DR15 heart grafts were tested for reactivity to various antigens of interest. During both acute (5/13) and chronic rejection (6/13), all recipients developed Tv−DTH responses to ColV, with low responses seen toward vimentin and donor antigen. (ii) Mice that rejected the grafts acutely (<25 d) developed immune responses to the DR1−binding peptide p629 but not to the DR15 epitope p1049. However those rejecting chronically responded to the donor DR15–restricted peptide p1049, as well as the DR1/15 cross-reactive peptides p799 and p1439 (*Unpaired t−tests *P* < 0.05). Tv−DTH, trans-vivo delayed type hypersensitivity.

To determine the influence of HLA−DR15 on transplant outcomes and the development of ColV reactivity, we transplanted cardiac grafts from DR15 donors into DR1 recipient mice. No immunosuppression was administered to any of the recipients. Recipient mice were sacrificed when the grafts stopped beating. While 40% of the grafts stopped beating between days 20–25 post-transplant (acute rejectors), the remaining grafts continued to beat, albeit at a reduced strength. These long−term survivors were sacrificed between days 80–100 post-transplant (Figure 3A). The syngeneic control grafts all survived, albeit with a decreasing graft score (data not shown).

Tv−DTH analysis of cells from both groups, acute and chronic rejectors, revealed low responses to the donor antigen and the heart−associated self−antigen vimentin but strong responses to ColV (Figure 3Bi). However, the fine specificity of the latter response, to ColV peptides, was different over time (Figure 3Bii). Acute rejectors responded only to the recipient DR1−restricted ColV peptide p629 but not to the DR15−restricted peptide p1049 or the DR1/15 cross-reactive peptides, p799 and p1439 (Figure 3Bii). In contrast, mice with long−surviving grafts developed responses not only to p629 but also to the DR15−restricted peptide p1049, as well as to the DR1/15 cross-reactive peptides, p799 and p1439 (Figure 3Bii). No responses to ColI or to the IA^b^ (and DQ)−restricted peptide p599 were observed.

**FIGURE 4.**
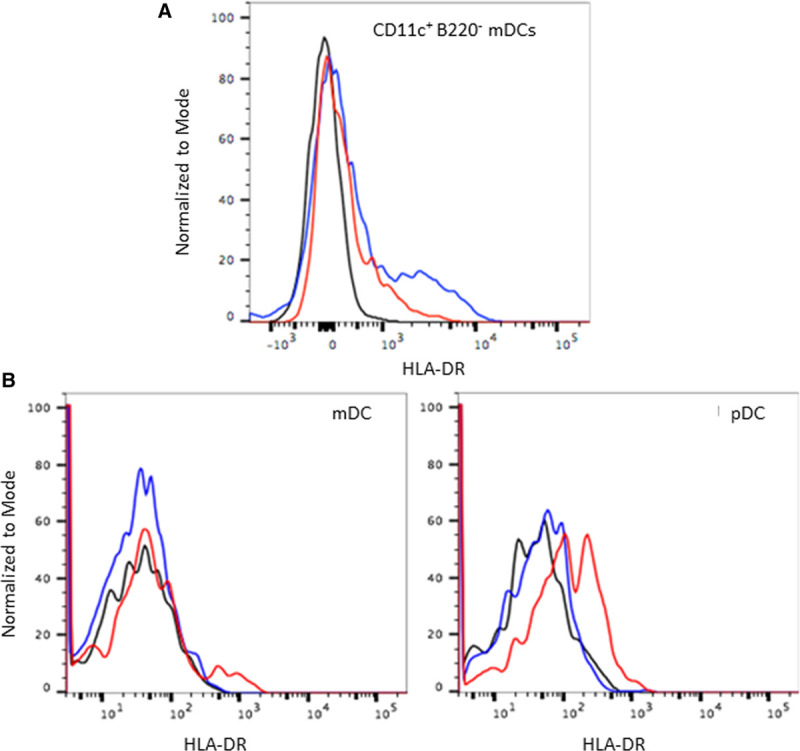
Acquisition of donor HLA−DR by recipient APCs. Naïve WT B6 mice were used as recipients of cardiac grafts from HLA−DR15 transgenic mice. HLA−DR expression in the recipients was analyzed using a pan DR antibody. A, Acquisition of donor HLA−DR on *graft−infiltrating dendritic cells* at day 14 post-transplant: B6 recipient of a DR15 graft was sacrificed at day 14 post-transplant. Live CD45^+^ graft−infiltrating cells were analyzed for donor HLA−DR expression. HLA−DR expression was noted on the graft−infiltrating dendritic cells, as seen by the small shift in the histogram (red) as compared with the negative control (black). Red line, Test DR15 to B6; Blue, WT DR15^+^ cells; Black, WT B6 cells. B, Acquisition of donor HLA−DR on *lymph node DC* at day 35 post-transplant: B6 recipient rejected the DR Tg heart at day 35 post-transplant. Lymph node cells were analyzed for HLA−DR expression. HLA−DR was expressed on both mDCs and pDCs, but not on monocytes (data not shown). Red line, Test DR15 to B6; blue, WT B6 cells; black, HLA−DR PE FMO. DC, dendritic cells.

We previously reported MHC−class II acquisition in newborns, in both mDC and pDC, driven by uptake of EVs released from the maternal DC.^12^ In order to determine whether this mechanism can also operate following organ transplantation, HLA−DR15 transgenic heart allografts were transplanted into WT B6 recipients. One recipient was euthanized at day 14 post-transplant, and infiltrating cells procured by collagenase digestion of the heart allograft were analyzed for HLA−DR expression. Clear expression of HLA−DR on CD45^+^ IAb^+^ CD11c^+^B220^−^ mDCs was observed in the graft−infiltrating leukocytes (Note: The expression level was clearly less than that seen on mDCs from the DR−transgenic donor, consistent with exosome−based acquisition by B6 host DC). A second recipient was followed until the graft stopped beating at day 35 post-transplant. At this time, the same low level of donor HLA−DR expression was noted on both mDCs and pDCs in the spleen and the lymph nodes of the recipient mice (Figure 4B). No HLA−DR expression was seen on monocytes (data not shown).

## DISCUSSION

The immune response (IR) is shaped by the MHC antigens inherited from mother and father and by maternal microchimerism−derived noninherited maternal antigens (NIMA).^22−26^ In postpartum women, and younger siblings, paternal HLA due to persistent fetal microchimerism can also have consequences for autoimmunity later in life.^22−24^ The present study demonstrates that MHC class II antigens arising from lung or heart transplant donor organs can also impose new self−recognition patterns upon graft recipients. These findings expand upon the discoveries by Benichou^27^ and Morelli^28^ that acute allograft rejection is caused by the “crossdressing” of host DC by EVs derived from rare “allo” DCs within the allograft. If the graft is spared (eg, by abortive acute rejection, or clinically by IS drug therapy), the long−term consequence of such EV crossdressing of host DC appears to be that the host begins to “see” certain self−antigens differently. We previously reported that specific donor HLA−DR antigen types play an important role in chronic rejection following lung transplantation, conferring susceptibility (DR15) or resistance (DR7) to the development of chronic rejection (BOS).^11^ In the present studies, we sought to further understand the mechanistic details facilitating such divergent outcomes post-transplantation, using PBMCs from HLA−DR mismatched human lung transplant recipients, and splenocytes from an HLA−DR−transgenic murine heart transplant model.

We found that DR15−restricted responses routinely develop in DR15^neg^ individuals >1.5 years after having received a DR15^+^ lung transplant. Although early (<3 mo post-transplant) human lung transplant blood samples were not tested, spleen samples from a DR Tg mouse heart transplant model suggest that this change, from self−DR−restriction to donor DR restriction of the anti−Col V response, happens gradually, taking hold after the period of acute rejection has passed. The current results reinforce previously published single patient data^7^ and complement the observations that HLA−DR15^neg^ recipients of DR15^+^ lung allografts are more likely to develop early (<1 y) or late (>1 y) chronic rejection following organ transplantation.^11,29^

Both the HLA−DR−transgenic strains used here, as well as B6 mice respond naturally in a self class II−restricted manner to ColV α1 chain fragments (Figure S1, SDC, http://links.lww.com/TXD/A285).^9,10,16^ Importantly, the naïve T cells of these mice, as previously reported,^8^ did not respond to 15−mer peptides of ColV because they require a second signal, provided by binding of intact ColV or larger fragments of the ColV α1 chain to LAIR1.^9^ Once sensitization has occurred, as in the case of lung or heart transplantation, this requirement ceases and response to a ColV peptide, either self− or donor−DR restricted, can proceed.

Patients who receive lung allografts from DR15 donors have increased susceptibility to the development of BOS. However, grafts from DR7^+^ donors were significantly resistant to BOS.^11^ While as of yet we lack mechanistic data to understand DR7^+^ donor−based protection, in the present study, we found a hint of how a patient might compensate for the greater immunogenicity of DR15^+^ grafts. In our small sample, the 3 DR15^neg^ recipients whose lymphocytes responded to the DR15−restricted peptide p1049, but did *not* regulate such responses, all showed relatively poor clinical outcomes. In contrast, the 4 patients that developed regulation, including *late* regulation in the case of L86, to the donor−DR15−restricted peptide of ColV, were all stable and doing well post-transplant (Table 1).

While all 7 patients in our study developed a response to the donor (DR15)−specific ColV peptide post-transplant, 4/7 also developed regulation of this response by either TGF−β (conventional) and IL−35 (EV−based^21^) cytokines, or both. The role of IL−35 in regulation was previously reported in monkey,^30^ human,^31^ and mouse^32^ immune tolerance studies. Thus, the present report documents a major EV influence on both antigen presentation (DR15 acquisition from the graft by host DC) and regulatory T function (IL−35 “exokine”) in clinical transplantation.

In conclusion, our data confirm the previous observation made in a single lung transplant patient, that the donor HLA−DR changes the host response to a key self−antigen.^7^ The current study also provides a possible explanation for how the donor HLA−DR15 type can promote chronic rejection and thereby diminish long−term graft survival,^11^ unless de novo immune responses to ColV “self” peptides become regulated (Table 1). This explanation is different from the classic test of “allo−antigenicity”, that is, the ability of an HLA difference between host and donor to promote acute rejection. During acute rejection by the HLA−DR1/A2 transgenic mice, the donor heart HLA−DR15 served as the target of CD4 T−cell “direct” alloresponses. At this early time point, the “indirect” self−antigen recognition pathway also functioned well, in that peptides of the self−antigen ColV, likely exposed on the allograft, were recognized entirely based on the *inherited* HLA−DR1 antigens of the recipient (Figure 3). However, once the acute rejection period had passed, grafts still surviving begin to reshape the recipient’s immune response, and now the *donor* HLA−DR15 began to restrict the anti−ColV response of the host (Figure 3B). The mechanism behind such a phenomenon is consistent with the semi-direct (or semi-indirect?) pathway, that is, acquisition of donor class II molecules after exosome secretion by the graft and uptake and reexpression by host DC (Figure 4). However, this “nonclassical” exosome−based mechanism, while clearly demonstrated in mice,^12,27,28^ is still unproven in man, and will require further verification.

## ACKNOWLEDGMENTS

The authors thank John Kernien for helping with back−crossing the DR15 transgenic mice.

We would also like to acknowledge Mehgan Holland and Don Hawes in the Cardiopulmorary Transplant coordination of UW Hospital for their invaluable help.
